# Sun Exposure and Protection Index (SEPI) and Self-Estimated Sun Sensitivity

**DOI:** 10.1007/s10935-018-0520-0

**Published:** 2018-08-16

**Authors:** Karin Widemar, Magnus Falk

**Affiliations:** Division of Community Medicine, Primary Care, Department of Medicine and Health Sciences, Linköping University, 581 83 Linköping, Sweden

**Keywords:** Skin cancer, Sun habits, Sun protection, Ultraviolet exposure, Questionnaire

## Abstract

The incidence of skin cancer is increasing worldwide, mostly because of increasing exposure to ultraviolet (UV) radiation from the sun. The Sun Exposure and Protection Index (SEPI) questionnaire, developed in Linköping and validated in Sweden and Australia, is used to map sun habits, sun protection behaviour, and readiness to increase sun protection. We sought to examine differences in sun habits or sun protection behaviour and propensity to increase sun protection, based on SEPI as related to self-estimated skin UV sensitivity according to the Fitzpatrick classification. The study population comprised students at Linköping University, who were asked to complete the SEPI questionnaire. We examined differences in sun habits and sun protection behaviour according to skin type and gender. Individuals with lower UV sensitivity had significantly riskier sun habits and sun protection behaviour and were significantly less likely to increase sun protection. Women spent significantly more time tanning than men, more time in the midday sun, used sunscreen more frequently, and were more likely to seek the shade for sun protection. Individuals with higher UV sensitivity were significantly more likely to increase sun protection; individuals with low UV sensitivity tended to have a riskier attitude to sunbathing. In conclusion, self-estimated skin type and gender are important factors influencing sun exposure habits and sun protection behaviour.

## Introduction

The Sun Exposure and Protection Index (SEPI) questionnaire for scoring of sun habits and readiness to increase sun protection was recently developed and has been validated in two different ultraviolent radiation (UVR) environments (Sweden and Australia; Detert, Hedlund, Anderson, Rodvall, Whiteman, Festin, & Falk, 2015; Falk & Anderson, 2014). The instrument builds on two types of behaviour, namely present sun exposure habits and propensity to increase sun protection. The purpose of the instrument is to identify both individuals with risky sun habits and those more prone to actually increase their use of sun protection. The instrument has high repeatability and an overall acceptable level of validity when compared to previously validated measures of similar content, and is suggested as a potentially useful tool to communicate sun protection advice, as well as targeting those with the highest need for advice (Detert et al., 2015; Falk & Magnusson, 2011). It may also be used to monitor the effects of preventative interventions, e.g., in research studies.

As the incidence of skin cancer has increased dramatically worldwide during the past decades (Erdei & Torres, 2010; Gruber & Armstrong, 2006; Lomas, Leonardi-Bee, & Bath-Hextall, 2012; Rigel, 2008; Stewart & Wild, 2014), there is a pressing need for increased preventive measures. This is true not only for malignant melanoma (MM), the most lethal type of skin cancer, but also for non-melanoma skin cancer (NMSC). Australia has the highest incidence of both MM and NMSC, whereas countries in Africa have the lowest incidence (Erdei & Torres, 2010; Gruber & Armstrong, 2006; Lomas, Leonardi-Bee, & Bath-Hextall, 2012). In Sweden, the incidence has more than doubled during the past two decades and MM is now the 6th and 5th most common cancer type in men and women, respectively. NMSC is the second most common cancer type in both sexes (National Board of Health and Welfare, 2015). There are a number of possible reasons contributing to the increase in skin cancer incidence: increasing life expectancy accompanied by an increasing proportion of elderly in the population; better awareness among the public; and, not least, more pronounced sun-seeking behaviour in general (Erdei & Torres, 2010; Rigel, 2008). The change in sun-seeking behaviour to a great extent derives from the notion that tanned skin is more appealing and a sign of health and well-being, holidays in warm and sunny locations have become more common and affordable, tanning beds are more frequently used, and smaller clothing and swimwear in warm temperatures have become increasingly popular (Erdei & Torres, 2010; Lautenschlanger, Wulf, & Pittelkow, 2007; Norval, Lucas, Cullen, de Gruiji, Longstreth, Takizawa et al., 2011).

Exposure to UV radiation (UVR) is the most well-known risk factor for skin cancer by causing damage to bio-molecular structures at the DNA level (Rigel, 2008). Therefore, avoidance of UVR is the most effective way to minimize the risk for skin cancer (Gruber & Armstrong, 2006, Rigel, 2008; Stewart & Wild, 2014). Because many of the other known risk factors are by nature impossible to alter (e.g., skin pigmentation, family history, nevi count), taking precautions in the sun is even more important for some individuals. In order to attempt to prevent skin cancer, we need to identify people with risky sun habits and provide them with adequate information on how to properly protect themselves against solar radiation. Various interventions to promote adequate sun protection have been explored in numerous studies and in different population groups, with varying degrees of success (Sánchez, Nova, Rodriguez-Hernandez, Medina, Solorzano-Restrepo et al., 2016; Sandhu, Elder, Patel, Saraiya, Holman et al., 2016; Wu, Aspinwall, Conn, Stump, Grahmann, & Leachman, 2016). Of note is that different measures are used in most studies, and that no gold standard for assessment of sun exposure and protection practices (to date) exists. However, independent of the method used, the identification of specific risk groups and/or individuals more prone to increase sun protection (thereby more likely to react positively to sun protection advice if given), appears to be crucial for a successful intervention. Identification and development of viable and easily accessible tools to detect high risk individuals who would benefit from tailored sun protective advice plays an important role in that respect.

The most common ways of protecting the skin from UVR are staying in the shade or indoors during the middle of the day, wearing protective clothing and a wide-brimmed hat, and applying sunscreen. Sun-seeking behaviour, as well as propensity to increase sun protection, vary by different age groups, gender, and level of education. Females in general sunbathe and use sunbeds more than men, but women, on the other hand, tend to use more sunscreen and to have a higher propensity to increase their use of sunscreen (Antonov, Hollunder, Schliemann, & Elsner, 2016; Boldeman, Bränström, Dal, Kristjansson, Rodvall, Jansson et al., 2001; Falk & Anderson, 2013). People over 65 years of age have the lowest level of sun exposure but also have the lowest readiness to increase sunscreen use (Falk & Anderson, 2013; Goulart & Wang, 2010). People with a higher level of education use more sunscreen and are more likely to increase sunscreen use than people with a lower level of education (Falk & Anderson, 2013). The incidence of NMSC, mainly squamous cell carcinoma, is higher among men, which could be explained partly by occupational and recreational factors; males more often work outdoors and practice outdoor recreational activities, exposing a larger area of skin than women, and they are less likely to use sunscreens (Lautenschlanger, Wulf, & Pittelkow, 2007; Norval et al., 2011). The incidence of MM is roughly the same for both women and men (Lomas, Leonardi-Bee, & Bath-Hextall, 2012; Norval et al., 2011).

The Fitzpatrick skin type classification is a commonly used method to measure self-estimated UV sensitivity. It consists of a scale with six skin type categories, according to the tendency to burn and tan, as follows (Fitzpatrick, 1988):

- Skin type I: always burns, never tans
- Skin type II: usually burns, tans minimally
- Skin type III: sometimes mild burn, tans uniformly
- Skin type IV: rarely burns, always tans well
- Skin type V: moderately pigmented brown skin, very rarely burns, tans very easily
- Skin type VI: deeply pigmented dark brown to black skin; never burns, tans very easily

In previous studies, self-estimated skin sensitivity using the Fitzpatrick classification has been shown to be poorly correlated with actual skin sensitivity, measured by UV phototesting (Baron, Stern, & Taylor, 1999; Boldeman, Dal, Kristjansson, & Lindelöf, 2004). Self-estimated skin sensitivity, on the other hand, is more highly correlated with individuals’ behaviour in the sun than actual UV sensitivity (Falk, 2011, 2014).

The Sun Exposure and Protection Index (SEPI) questionnaire, which rates sun habits and readiness to increase sun protection, was recently developed and has been validated in two different UVR environments (Sweden and Australia). In contrast to many other instruments addressing sun exposure habits, which are rather extensive and time consuming, the SEPI is quite short, taking only a few minutes to complete (Detert et al., 2015). It consists of two parts, the first of which includes eight questions based on a 5-point Likert scale (0 = low risk behaviour to 4 = high risk behaviour) regarding sun habits and sun protection behaviour, thus resulting in a total score of 0–32 points, for which a high score reflects a more risky/less protective behaviour in the sun. The second part maps readiness to increase sun protection, based on the transtheoretical model of behaviour change (TTM), and consists of five questions, also scored 0–4 points, in this case reflecting decreasing propensity to increase sun protection, resulting in a total score of 0–20 points. The TTM is well established in behavioural medicine (including sun habits and skin cancer prevention) and describes behaviour change as a process over time through six stages: precontemplation, contemplation, preparation, action, maintenance and termination (Prochaska, 2013). Taken together, a high total score on both parts of the SEPI typically reflects an individual with risky UV exposure habits, also with low propensity to change it. The individual question items for both SEPI parts are displayed in Tables 1 and 2. The SEPI, with its two scores, can be used as a tool for individualized UV exposure risk assessment and risk communication in a clinical setting, as well as an instrument for mapping sun exposure and protection on a population level, e.g., in research studies, and to evaluate the effect of a given intervention. Since the instrument was developed and validated recently, little is known about its relation to self-estimated UV sensitivity as assessed by the traditional Fitzpatrick classification.

**Table 1:** The distribution of Likert scale responses on sun exposure habits and sun protection behaviour (SEPI part I) according to self-estimated skin type and gender (0 = low risk to 4 = high risk points for each individual question item, and 0–32 points for the total score)

	Intentional tanning	Occasions with sunburn	Time spent in the midday sun	Sun vacation abroad	Sunscreen use	Protective clothing use	Protective headwear use	Staying in the shade	Total score
*Skin type* (*Fitzpatrick*)									
Skin type I (*n* = 18)									
Mean	0.78	1.72	1.67	1.06	1.17	2	2.83	1.39	12.61
Median	1	2	1.5	1	1	1	3	1	12.5
Skin type II (*n* = 130)									
Mean	1.78	1.4	1.92	1.45	1.33	2.31	2.52	2.1	14.8
Median	2	1	2	1	1	2	3	2	15
Skin type III (*n* = 213)									
Mean	2.24	1.06	2.25	1.62	1.3	2.68	2.62	2.4	16.16
Median	2	1	2	2	1	3	3	2	16
Skin type IV (*n* = 41)									
Mean	2.24	0.44	2.66	1.95	2.15	3.2	3.02	2.68	18.34
Median	2	0	3	2	2	3	3	3	19
Skin type V (*n* = 8)									
Mean	2.13	0.25	2.25	2.25	2.5	3.63	3.63	2.88	19.5
Median	2	0	2	2	2.5	4	4	3	19
*p*	< *0.001*	< *0.001*	< *0.001*	< *0.001*	< *0.001*	< *0.001*	*0.003*	< *0.001*	< *0.001*
*Gender*									
Female (*n* = 202)									
Mean	2.37	1.21	2.39	1.65	1.18	2.61	2.66	2.42	16.49
Median	2	1	2	2	1	3	3	2.5	17
Male (*n* = 208)									
Mean	1.69	1.03	1.94	1.52	1.63	2.59	2.64	2.18	15.23
Median	2	1	2	1	1	3	3	2	15
*p*	< *0.001*	*0.043*	< *0.001*	0.055	< *0.001*	0.668	0.924	*0.007*	*0.002*

*p* values are based on Kruskal–Wallis analysis (skin type), Mann–Whitney *U* test (gender) and median test analysis (total scores). *p* values less than 0.05 are shown in italics

**Table 2:** The distribution of responses on readiness to increase sun protection, based on the TTM (SEPI part II), according to self-estimated skin type and gender (0–4 points for each individual question item, and 0–20 points for the total score)

	Giving up sunbathing	Sunscreen use	Clothes for sun protection	Headwear for sun protection	Staying in the shade	Total score
*Skin type* (*Fitzpatrick*)						
Skin type I (*n* = 18)						
Mean	1.22	0.33	1.56	2.39	0.83	6.33
Median	0.5	0	1	3	0	5
Skin type II (*n* = 130)						
Mean	2.54	0.62	2.19	2.12	1.75	9.22
Median	3	0	3	3	2	9
Skin type III (*n* = 213)						
Mean	3.01	0.62	2.7	2.22	2.32	10.88
Median	3	0	3	3	3	11
Skin type IV (*n* = 41)						
Mean	3.27	1.41	3.24	3.05	3	13.98
Median	4	1	3	3	3	15
Skin type V (*n* = 8)						
Mean	3.63	1.62	3.13	3.13	2.63	14.13
Median	4	1.5	3	3	3	14.5
*p*	< *0.001*	*0.001*	< *0.001*	< *0.001*	< *0.001*	< *0.001*
*Gender*						
Female (*n* = 202)						
Mean	2.8	0.51	2.55	2.29	2.15	10.3
Median	3	0	3	3	3	11
Male (*n* = 208)						
Mean	2.85	0.9	2.55	2.3	2.14	10.75
Median	3	0	3	3	3	11
*p*	0.334	*0.002*	0.404	0.553	0.741	0.31

*p* values are based on Kruskal–Wallis analysis (skin type), Mann–Whitney *U*-test (gender) and median test analysis (total scores). *p* values less than 0.05 are shown in italics

In this study, we investigated whether there were any differences in sun habits, sun protection behaviour or propensity to increase sun protection, as assessed by SEPI, with regard to self-estimated skin UV sensitivity according to the Fitzpatrick classification.

## Methods

### Study Population

The study was performed in Linköping, Sweden, during September to October 2015. In order to secure a heterogeneous sample, the population consisted of students from different education programmes at Linköping University. These programmes, included medicine, physiotherapy, psychology, applied physics and electrical engineering, mechanical engineering, industrial engineering and management, commercial and business law and information systems.

We administered surveys during a 5-week period by visiting course lectures and classes at the selected programmes, after having received permission from the lecturer to distribute the questionnaire. The students received a short verbal introduction with information about the study and were then handed a consent form, a written information sheet about the study, and the questionnaire. The students were given the option of completing the questionnaires at that time or later at home and then mailing it in a pre-paid envelope.

The inclusion criteria for the participants were age ≥ 18 years and being a registered student at any of the university programmes listed above. No remuneration was given for participation. All study procedures were approved by the Regional Ethical Review Board in Linköping.

### Questionnaire

In addition to the two parts of SEPI, the survey also included questions concerning respondents’ age, gender, university programme, previous history of skin cancer or skin cancer in the family (first-degree relative), self-estimated UV sensitivity according to the Fitzpatrick classification, and a brief mapping of attitudes towards sunbathing, based on 0–4 point Likert scale scores (0 = low risk attitude, 4 = high risk attitude), except for one question that was scored 0–3 points (see Table 3).

**Table 3:** The distribution responses on attitude towards sunbathing according to self-estimated skin type and gender

	Fondness of sunbathing^a^	Pros and cons of sunbathing^a^	Benefit and harm of sunbathing^a^	Self-estimated risk of skin cancer^a^	Importance of tanned skin in the summer^b^
*Skin type* (*Fitzpatrick*)					
Skin type I (*n* = 18)					
Mean	1.56	0.83	0.61	2.33	0.61
Median	2	0.5	0.5	2	0.5
Skin type II (*n* = 130)					
Mean	2.29	1.57	0.88	2.31	1.34
Median	2	1	1	2	1
Skin type III (*n* = 213)					
Mean	2.79	1.92	1.07	2.23	1.6
Median	3	2	1	2	2
Skin type IV (*n* = 41)					
Mean	2.78	2.05	1.2	2.17	1.8
Median	3	2	1	2	2
Skin type V (*n* = 8)					
Mean	2.88	2.5	1.88	2.88	1.5
Median	3	2.5	2	3	1
*p*	< *0.001*	< *0.001*	*0.001*	*0.002*	< *0.001*
*Gender*					
Female (*n* = 202)					
Mean	2.82	1.72	0.81	2.05	1.7
Median	3	2	1	2	2
Male (*n* = 208)					
Mean	2.35	1.85	1.22	2.47	1.28
Median	2	2	1	2	1
*p*	< *0.001*	0.214	< *0.001*	< *0.001*	< *0.001*

*p* values are based on Kruskal–Wallis analysis (skin type) and Mann–Whitney *U* test (gender). *p* values less than 0.05 are shown in italics

^a^Values on a 0 (low risk) to 4 (high risk) Likert scale

^b^Values on a 0–3 Likert scale

### Statistical Analysis

We calculated differences in outcome between different skin types and gender for SEPI Part I (0–32 points) and Part II (0–20 points), as well as for the remaining variables. To investigate statistical differences in median values of the total scores of SEPI Parts I and II, we used the non-parametric independent samples median test; for skin type we used the Kruskal–Wallis analysis, and for gender, we used the Mann–Whitney *U*-test. A confidence interval of 95% was set as statistically significant for all analyses. We calculated the correlation between SEPI Parts I and II using linear regression. A correlation coefficient of 0.5–1 was interpreted as a strong correlation. For all analyses, we used the statistical software package SPSS (IBM SPSS Statistics 23, IBM, New York, USA).

## Results

We invited a total of 566 students to participate, of whom 443 completed the questionnaire. Of these, eleven (2.5%) were excluded because their consent forms were missing and 22 (5%) were excluded due to incomplete questionnaires. Of the remaining 410 participants, 202 (49.3%) were female. Their ages ranged between 18 and 51 years, with a mean of 22 years (*SD* ± 3.7). Of the 410 participants, 40 (9.8%) were studying medicine, 50 (12.2%) were studying physiotherapy, 56 (13.7%) psychology, 50 (12.2%) applied physics and electrical engineering, 41 (10%) mechanical engineering, 77 (18.8%) industrial engineering and management, 29 (7.1%) commercial and business law, and 67 (16.3%) were studying information systems. The distribution of participants according to self-estimated skin type was as follows: 18 (4.4%) had skin type I, 130 (31.7%) had skin type II, 213 (52%) had skin type III, 41 (10%) had skin type IV and 8 (2%) had skin type V. None of the participants reported having skin type VI. None of the participants reported having a history of skin cancer, and only 33 (8%) reported having a first-degree relative who had had skin cancer.

### Sun Habits and Sun Protection Behaviour (SEPI Part I)

Table 1 presents the distribution of responses to the individual questions in SEPI Part I exploring sun exposure habits and sun protection behaviour, and the total SEPI Part I score with regard to self-estimated skin type and gender; mean and median values are provided with *p* values based on Kruskal–Wallis analysis (skin type), Mann–Whitney *U*-test (gender) and median test analysis (total scores).

**Fig. 1:**
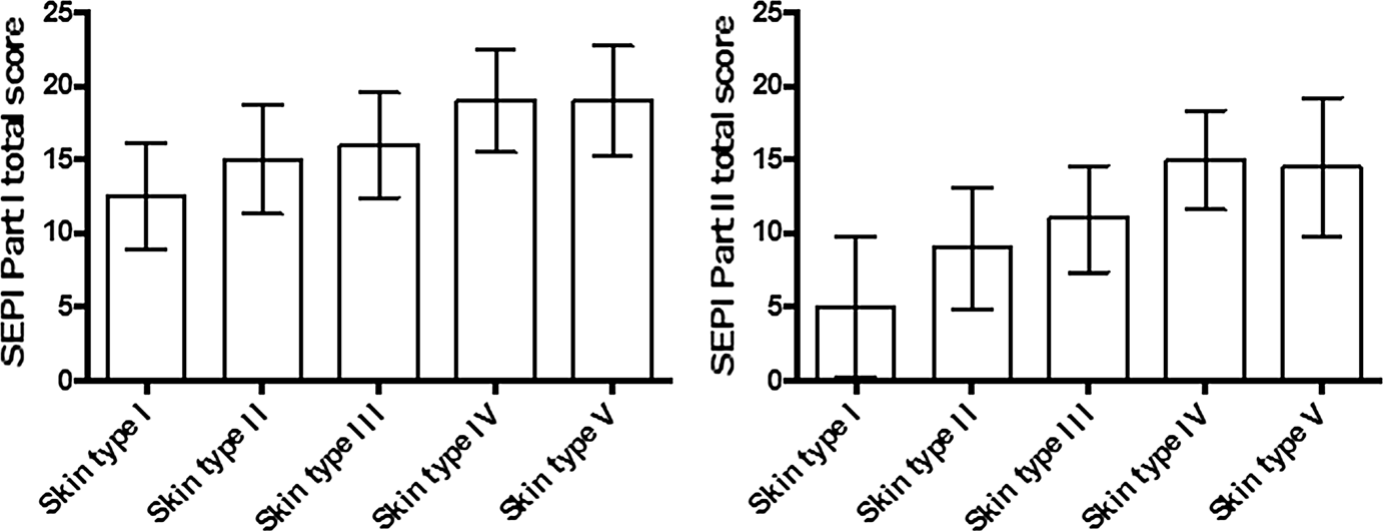
The median values of the total score from SEPI parts I and II, according to skin type. A high SEPI score in part I (left diagram) indicates a high risk behaviour, and in part II (right diagram) a low propensity to change it, whereas skin types I–V represent decreasing self-estimated UV-sensitivity. Error bars show the standard deviations

Individuals with a reported high UV sensitivity had a lower mean and median score on every individual question except for the question regarding occasions with sunburn, for which they had the highest mean and median score, reflecting their higher UV sensitivity. In contrast, individuals with a reported low UV sensitivity had higher mean and median scores on every question except for occasions with sunburn. However, all skin types had a low mean and median score for the question regarding sunscreen use. The question regarding protective headwear use scored high among all skin types. The median value for the total score was higher for individuals with a lower self-estimated UV sensitivity (*p* < 0.001; Fig. 1).

The results show that women reported spending more time tanning than men (*p* < 0.001), as well as more time in the midday sun (*p* < 0.001) and had more occasions with sunburn (*p* < 0.05). However, women also reported using sunscreen more frequently (*p* < 0.001) and to be more likely to protect themselves by staying in the shade (*p* < 0.01). Questions regarding sun vacation abroad, protective clothing and protective headwear use showed no significant differences between genders. Women had a significantly higher median total score than men (*p* < 0.005), thus reflecting an on average more risky behaviour in the sun.

### Readiness to Increase Sun Protection (SEPI Part II)

Table 2 presents the distribution of responses to the questions in SEPI Part II, exploring readiness to increase sun protection, as well as the total SEPI Part II score, with regard to self-estimated skin type and gender; mean and median values are presented with *p* values based on Kruskal–Wallis analysis (skin type), Mann–Whitney *U*-test (gender) and median test analysis (total scores).

Individuals with a reported high UV sensitivity had a lower mean and median score for every question as compared with individuals with a reported low UV sensitivity. Only skin type I was associated with low scores on the questions regarding giving up sunbathing (*p* < 0.001), protective clothing (*p* < 0.001) and staying in the shade (*p* < 0.001), whereas individuals reporting other skin types were in general more reluctant to increase sun protection. The mean and median scores for the question on sunscreen use were low for all skin types, whereas all skin types scored high on the question on protective headwear use. The median value for the total score was higher in individuals with low compared to high self-estimated UV sensitivity (*p* < 0.001), as demonstrated in Fig. 1.

Although women were shown to use sunscreens more frequently in SEPI Part I, women in SEPI Part II were still more likely to increase sunscreen use than men (*p* < 0.005). Questions regarding giving up sunbathing, protective clothing, protective headwear use and staying in the shade showed no significant differences between genders.

### Attitudes Towards Sunbathing

Table 3 presents the results from the questions on attitudes towards sunbathing according to self-estimated skin type and gender; the mean and median values are presented with *p* values based on Kruskal–Wallis analysis (on skin type groups) and Mann–Whitney *U*-test (on gender).

Participants with lower UV sensitivity had in general a more positive attitude towards sunbathing than those with higher UV sensitivity (*p* < 0.001). Participants with low UV sensitivity to a higher degree considered the advantages of sunbathing outweighed the disadvantages (*p* < 0.001) and that sunbathing was more beneficial than harmful (*p* < 0.001), whereas participants with high UV sensitivity tended to report the opposite. Individuals of all skin types reported that a slightly increased risk of developing skin cancer was a consideration. Participants with skin types III and IV thought it most important to get tanned in the summer, whereas those with skin type I reported this as least important (*p* < 0.001).

Women were in general more fond of sunbathing than men (*p* < 0.001), but also considered it to be more harmful than beneficial (*p* < 0.001) and that they had a greater risk of developing skin cancer (*p* < 0.001). Women in general also thought it was more important to get tanned in the summer (*p* < 0.001).

### Correlation Between SEPI Parts I and II

**Fig. 2:**
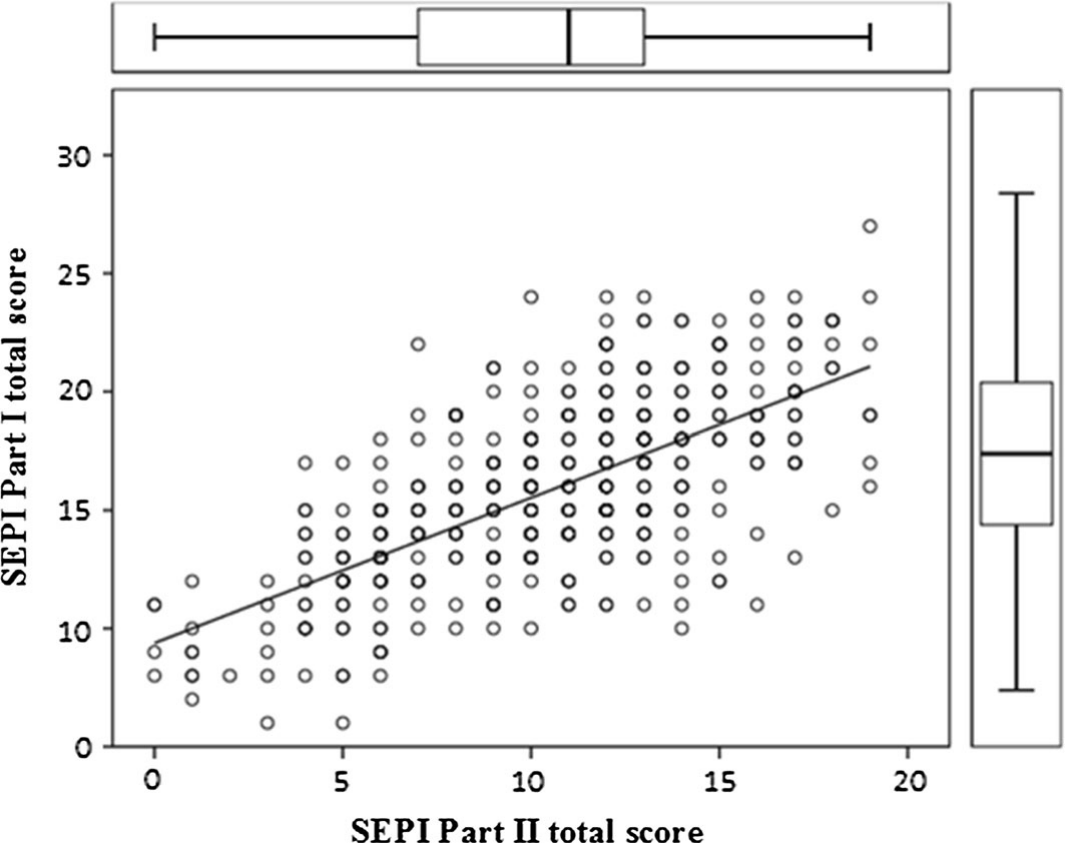
Scatterplot with regression line showing the relationship between the total scores of SEPI part I (0–32 points) and part II (0–20 points). Bolded circles represent plotted values consisting of more than one case. Standardized correlation coefficient 0.699 (unstandardized coefficient 0.615; *p* < 0.001). Adjusted r^2^ = 0.466

The correlation between total score in SEPI for Parts I and II was positive and high with a correlation coefficient (*r*) of 0.699 (*p* < 0.001) and adjusted *r*^2^ of 46%. The results from the analysis are displayed in Fig. 2.

## Discussion

Previous studies observing the relationship between self-estimated skin type and sun habits have shown that a sensitive skin type appears to be associated with a higher level of sun avoidance and protection (Falk, 2011, 2014; Falk & Anderson, 2013). We found that participants with high self-estimated UV sensitivity tended to protect themselves from the sun to a greater extent than did those with low self-estimated UV sensitivity. On the other hand, they also had a higher propensity (and were therefore more likely) to increase their use of protection. This seems reasonable because participants with high-sensitivity skin types reported the highest number of occasions with sunburn, which were likely to induce a subsequent perceived need to increase sun protection. In contrast, participants with risky sun habits appeared to be less willing to change their behaviour in a more sun-protective direction, as illustrated in Fig. 2. A probable reason for this finding might be that many of those with risky habits have a skin type less prone to sunburn and therefore do not see the benefit of increasing their use of protection. An instrument like SEPI in combination with assessment of skin type may constitute a constructive basis on which to communicate individualized sun protection advice, explaining why it might be beneficial to increase sun protection even if the individual perceives themselves to tolerate the sun well.

In concordance with previous studies (Falk, 2011; Falk & Anderson, 2013), our results highlight that individuals with lower UV sensitivity (skin types III–V) tended to spend more time tanning and more time on sun vacations abroad than those with higher UV sensitivity. Individuals with low as opposed to high UV sensitivity reported having a more positive attitude towards sunbathing, reported that the advantages and benefits of sunbathing outweighed the disadvantages and harm, and reported that it is more important to get a tan. A contributory reason that individuals with high UV sensitivity were less likely to spend time on sun vacations abroad may be the potential discomfort of getting sunburnt more easily when outdoors, making this recreational setting less desirable. In addition, it is possible that some individuals with darker skin types may have relatives in countries closer to the equator that they visit more regularly. The mean and median scores for the question about time spent in the midday sun did not vary across the skin type groups, which could reflect that, in practice, it may be difficult to schedule activities to avoid being outside at specific times during the day.

Women reported using sunscreen more frequently than men, which again is in concordance with previous studies (Antonov, Hollunder, Schliemann, & Elsner, 2016; Boldeman, Bränström, Dal, Kristjansson, Rodvall, Jansson et al., 2001; Falk & Anderson, 2013). However, women also reported spending more time tanning and more time in the midday sun, and experiencing a higher number of occasions with sunburn. This could explain why women were more willing to increase their use of sunscreen. Regarding attitudes towards sunbathing, our results indicate that women in general enjoy sunbathing more than men and think it more important to get tanned during the summer. However, women also reported that they considered sunbathing to be more harmful than beneficial and that they had a higher risk of developing skin cancer than men. This may reflect a plausible tension in the decisional balance between two contradictory sides of the matter: the wish to get tanned skin against the risk of getting sunburnt and a possible heightened risk of developing skin cancer, thus promoting a need for increase in sun protection use. The choice of sunscreen as the preferred protection could possibly reflect that it allows for some tanning of the skin, whereas other kinds of sun protection (such as long-sleeved clothing) prevents tanning to a greater extent.

This study has some methodological limitations. With regard to the study population, it is realistic to assume that individuals with a more pronounced interest in issues like sun exposure habits would be more likely to participate in the study, thus introducing a source of selection bias. However, the response rate was quite high (78%), suggesting that skin cancer is a subject of general interest among the public. The use of the Fitzpatrick classification as a measure of UV sensitivity could be questioned because previous studies have implied that it is poorly correlated with actual UV sensitivity measured by phototest (Baron, Stern, & Taylor, 1999; Boldeman, Dal, Kristjansson, & Lindelöf, 2004; Falk, 2011, 2014). A possible reason might be that individuals have difficulty judging their own sun sensitivity in relation to others, as well as their own behaviour in the sun, thereby having different perceptions of their reaction to UVR. Another reason may be that the Fitzpatrick classification takes into account both the tendency to burn and to tan, whereas phototesting only takes into account the tendency to burn (Baron et al., 1999; Boldeman et al., 2004; Falk, 2014; Fitzpatrick, 1988). However, although the Fitzpatrick classification is not completely reliable, it is widely used and accepted and has the advantages of being quick and easy to administer. With regard to generalizability of the study, a possible weakness was the skewing in the distribution of skin types towards the low end of the range, a distribution that in reality reflects the characteristics of the largely fair-skinned Scandinavian population.

We collected our survey data during September and October, i.e., during the post-sun season in Sweden. This strategy had both advantages and disadvantages. The advantage is that reported behaviour constitutes a summary of the past summer holiday period. On the other hand, the possibility of recall bias must be considered. Since sun exposure habits in Sweden comprise both domestic sun exposure mainly during June to August and travel to sunny resorts abroad all year round, errors related to recall bias are likely to occur also during other parts of the year, depending on an individual’s sun-seeking habits.

The use of SEPI scoring as a measurement variable appears to be an efficient and, according to previous instrument validation (Detert et al., 2015; Falk & Anderson, 2012), reliable method for mapping sun exposure habits, and well applicable to this type of study. It is not unlikely that the high response rate achieved in the study was in part associated with the ease with which the questionnaire could be completed. Also, the construction allows for interpretation of single behaviour items (e.g., sunscreen use), as well as the overall behaviour profile reflected by the total SEPI score.

In conclusion, self-estimated skin type, along with gender, appear to be important factors affecting sun exposure habits and sun protection behaviour. This may be of importance when communicating risk assessment and sun protection advice in the clinical setting, as well as in the design of future interventions aiming to reduce UV exposure in a targeted population.
